# Interleukin-23 Facilitates Thyroid Cancer Cell Migration and Invasion by Inhibiting *SOCS4* Expression via MicroRNA-25

**DOI:** 10.1371/journal.pone.0139456

**Published:** 2015-10-05

**Authors:** Zhidan Mei, Shiming Chen, Chen Chen, Bokui Xiao, Fen Li, Yongping Wang, Zezhang Tao

**Affiliations:** 1 Department of Otolaryngology Head and Neck Surgery, Remin Hospital of Wuhan University, Wuhan, 430060, Hubei Province, the People’s Republic of China; 2 Research Institute of Otolaryngology Head and Neck Surgery, Renmin Hospital of Wuhan University, Wuhan, 430060, Hubei Province, the People’s Republic of China; Peking University Cancer Hospital & Institute, CHINA

## Abstract

Interleukin–23 (IL–23) is a conventional proinflammatory cytokine that plays a role in tumor progression by inducing inflammation in the tumor microenvironment. However, the role of IL–23 in thyroid cancer migration and invasion remains unclear. In the present study, we observed that the treatment with IL–23, induced migration and invasion in human thyroid cancer cells. Additional data demonstrate that *SOCS4* negatively regulates IL-23-mediated migration and invasion. On investigating the mechanisms involved in IL–23 mediated migration and invasion, we observed that miR–25 promotes the migration and invasion of thyroid cancer cells by directly binding to the 3′-UTR of *SOCS4* that leads to the inhibition of *SOCS4*. In addition, we also demonstrated that IL–23 increases miR–25 expression levels, and overexpressed miR–25 is involved in IL-23-associated *SOCS4* inhibition and cell migration and invasion. Together, our data suggest that IL–23 induces migration and invasion in thyroid cancer cells by mediating the miR–25/*SOCS4* signaling pathway.

## Introduction

Thyroid cancer is a common type of endocrine malignancy that has shown a rapid increase in worldwide incidence during the past few decades [1]. Despite improvements in therapeutic strategies, some patients are difficult to treat and develop invasion and metastasis [2]. Therefore, it is essential to identify the molecular mechanisms underlying thyroid cancer invasion and metastasis. Recent reports demonstrated that inflammation is a strong promoter of carcinogenesis and malignancy in many forms of cancer [3,4]. Inflammation seems to be an important mediator for the development of cancer and provides the cancer cells a hospitable microenvironment [5]. Interleukin–23 (IL–23), a heterodimeric type 1 cytokine composed of the IL–12/p40 subunit and the specific p19 subunit, belongs to the interleukin–6 superfamily [6]. Previous studies have shown that IL–23 is associated with carcinogenesis as well as inflammation. High levels of IL–23 were found in human hepatocellular carcinoma, colorectal carcinoma, squamous carcinoma, and esophageal carcinoma [7–10]. Evidence suggests that IL–23 overexpression can induce metastasis in colorectal, lung, and oral cancer [7–10].

The suppressors of cytokine signaling (SOCS) are important negative feedback regulators of cytokine signaling [11]. The SOCS proteins are a family of 8 proteins (SOCS1-7 and a cytokine-inducible SH2-containing protein or CIS). Each SOCS protein contains a central SH2 domain that interacts with phosphorylated tyrosines [12]. SOCS proteins have been recently investigated for their role in the development of different cancers [13–18]. However, little is known about the role of SOCS4 in carcinoma, and their possible influence on tumor growth and malignancy.

MicroRNAs (miRNAs) are a species of small noncoding single stranded RNAs that play an important role in the development of different cancers by binding the 3′-untranslated region (3′-UTR) of targeted genes [19]. Aberrant miRNA expression has also been frequently reported in numerous tumors [20]. In recent years, multiple evidence point to a role for miRNAs in tumor cell biological processes, including cell proliferation, differentiation, migration, and invasion [21]. MicroRNA–25 belongs to the miR-106b-25 cluster that includes miR-106b, miR–93, and miR–25. MicroRNA–25 has been reported to be aberrantly overexpressed in several tumors, such as ovarian cancer, lung cancer, gastric cancer, and colorectal cancer [22–26]. Although the expression of miR–25 in different tumors has been described, a clear role for miR–25 in thyroid carcinoma remains unclear.

In this study, we demonstrate that IL–23 promotes thyroid cancer cell migration and invasion. We further demonstrate that IL–23 regulates the migration and invasion of thyroid cancer cells via a miR–25/SOCS4 signaling pathway.

## Materials and Methods

### Ethics statement

All participants gave written informed consent to participate in the study. The study was conducted according to the principles of the Declaration of Helsinki and approved by the Institutional Review Board of the Remin Hospital of Wuhan University, in accordance with its guidelines for the protection of human subjects.

### Samples and cases

Thyroid tissues were collected at the Remin Hospital of Wuhan University from February 2010 to February 2014. Tissue samples were cut into two parts, one was reviewed by two expert pathologists to verify the histologic diagnosis, the other immediately snap-frozen in liquid nitrogen, and stored in liquid nitrogen until RNA extraction. None of the patients had received any preoperative treatment. Tumors were staged according to the American Joint Committee on Cancer (AJCC) pathologic tumor-node-metastasis (TNM) classiication. The characteristics of patients are described in S1 and S2 Tables.

### Cell culture

Human thyroid cancer cell lines K1 (papillary) and WRO (follicular) were cultured in Dulbecco’s modified Eagle’s medium (DMEM) (Gibco BRL, Grand Island, NY) supplemented with 10% fetal bovine serum (Gibco BRL), 100 units/ml penicillin, and 100 μg/ml streptomycin sulfate. Cells were maintained at 37°C in a 5% CO_2_ incubator. All these cell lines were original purchased from cell bank of the Chinese Academy of Science, Shanghai.

### Reagents

Recombinant human IL–23 (rhIL–23) was purchased from R&D Systems (Minneapolis, MN). Monoclonal antibodies (Abs) against human SOCS4 and GAPDH were purchased from Sigma (St Louis, MO). Chemically synthesized miRNA mimics and miRNA inhibitors were purchased from Ambion (Austin, TX, USA). TRIzol, Lipfectamine–2000, Enzyme MIX were purchased from Invitrogen (Basel, Switzerland).

### Quantitative real time PCR

Quantitative real time PCR analysis was performed to determine mature miRNA and mRNA levels. For quantitative mature microRNAs detection, Total miRNAs were isolated using a mirVana miRNA isolation kit (Ambion), according to the manufacturer’s instructions. Total RNA (2 μg) was reversetranscribed with Bulge-Loop mature miRNA-specific reverse transcription primers (Ambion) and Moloney murine leukemia virus reverse transcriptase (Promega). Quantitative Real-time PCR-based quantification of miRNAs was performed using the miRNA analysis kits (Ambion), according to the manufacturer’s instructions. The levels of miRNAs were normalized to those of the internal control U6 snRNA. To detect cellular mRNAs, total RNA was isolated using TRIzol (Invitrogen, Basel, Switzerland). Cellular RNA samples were reverse-transcribed using random primers. Real-time PCR was performed using a LightCycler 480 (Roche) and the SYBR Green system (Applied Biosystems). GAPDH was amplified as an internal control. Primers used this study are listed in S3 Table and the SYBR green products verified by sequencing.

### Transwell assay

Cell migration and invasion assay were performed as described previously [27]. Briefly, cells were treated either with 50 ng/mL rhIL–23 or BSA buffer control for described time and dose and observed accordingly. The mean number of migrating and invading cells was expressed as a percentage relative to the control, which was designated as 100%.

### Wound closure assay

A wound was introduced on the confluent monolayer cells using a micropipette tip. Photographs were taken at 40 X magnification using phase-contrast microscopy immediately after wound incision and at selected timepoints. Wound closure was measured by calculating pixel densities in the wound area by Cella software (Olympus Biosystem Gmb, Hamburg, Germany) and expressed as percentage of wound closure of triplicate areas ± SD.

### MTT assay

The 3-(4,5-dimethylthiazole-2-yl)-2,5-diphenyltetrazolium bromide (MTT) assay was used in the evaluation of cells proliferation. Cells were seeded into 96-well plates at 5×10^3^ cells/well. Twenty-four hours later, MTT assay was conducted. Finally, the optical density was determined at 570nm using the ELISA plate reader (Model 550; Bio-Rad). At least three independent experiments were ensured.

### Transfection and luciferase reporter assay

Cells were seeded on 24-well or 6-well dishes, depending on the experiment, and were grown to the confluence reaching approximately 80–90% at the time of transfection. Cells were transfected using Lipofectamine 2000 (Invitrogen, Carlsbad, CA) according to the protocol provided by the manufacturer. A Renilla luciferase reporter vector pRL-TK was used as internal control. Luciferase assays were performed with a dual-specific luciferse assay kit (Promega, Madison, WI). Firefly luciferase activities were normalized on the basis of Renilla luciferase activities. All reporter assays were repeated for at least three times. Data shown were average values ± SD from one representative experiment.

### RNA interference

SOCS4 shRNA and irrelevant shRNA control (shRNA-control) were purchased from GenePharma (GenePharma, Shanghai) and prepared by ligation of the corresponding pairs of oligonucleotides to PGPU6/GFP/Neo. The target sequence can be found in S4 Table.

### Western blot analysis

Whole-cell lysates were prepared by lysing cells with PBS pH 7.4 containing 0.01% Triton X–100, 0.01% EDTA, and 10% protease inhibitor cocktail (Roche, Applied Science). Protein concentration was determined by the Bradford assay (Bio-Rad). The polypeptides from cell lysates were separated on SDS 12% polyacrylamide gels cross-linked with N,N -methylenebisacylamide, and transferred electrically to nitrocellulose membranes (Millipore, Billerica, MA). Nonspecific binding was blocked with 5% milk in PBST before adding primary antibodies used in this study. Horseradish peroxidase–linked anti-rabbit and anti-mouse antibodies (Sigma, St Louis, MO) were used as secondary antibodies. Protein levels were quantified by scanning blots on a Gel Doc EZ imager (Bio-Rad) and analysis with Quantity One 1D image analysis software 4.4.0 (Bio-Rad)

### Statistical analysis

Statistical analyses were performed using the GraphPad Prism 5 software (GraphPad Software, La Jolla, CA, USA). Parametric and nonparametric data were analyzed using a two-tailed t-test and the Mann–Whitney U test respectively. A value of P < 0.05 was considered statistically significant. Data are presented as mean±S.D. or mean±S.E.M.

## Results

### IL–23 promotes the migration and invasion of thyroid cancer cells

**Fig 1:**
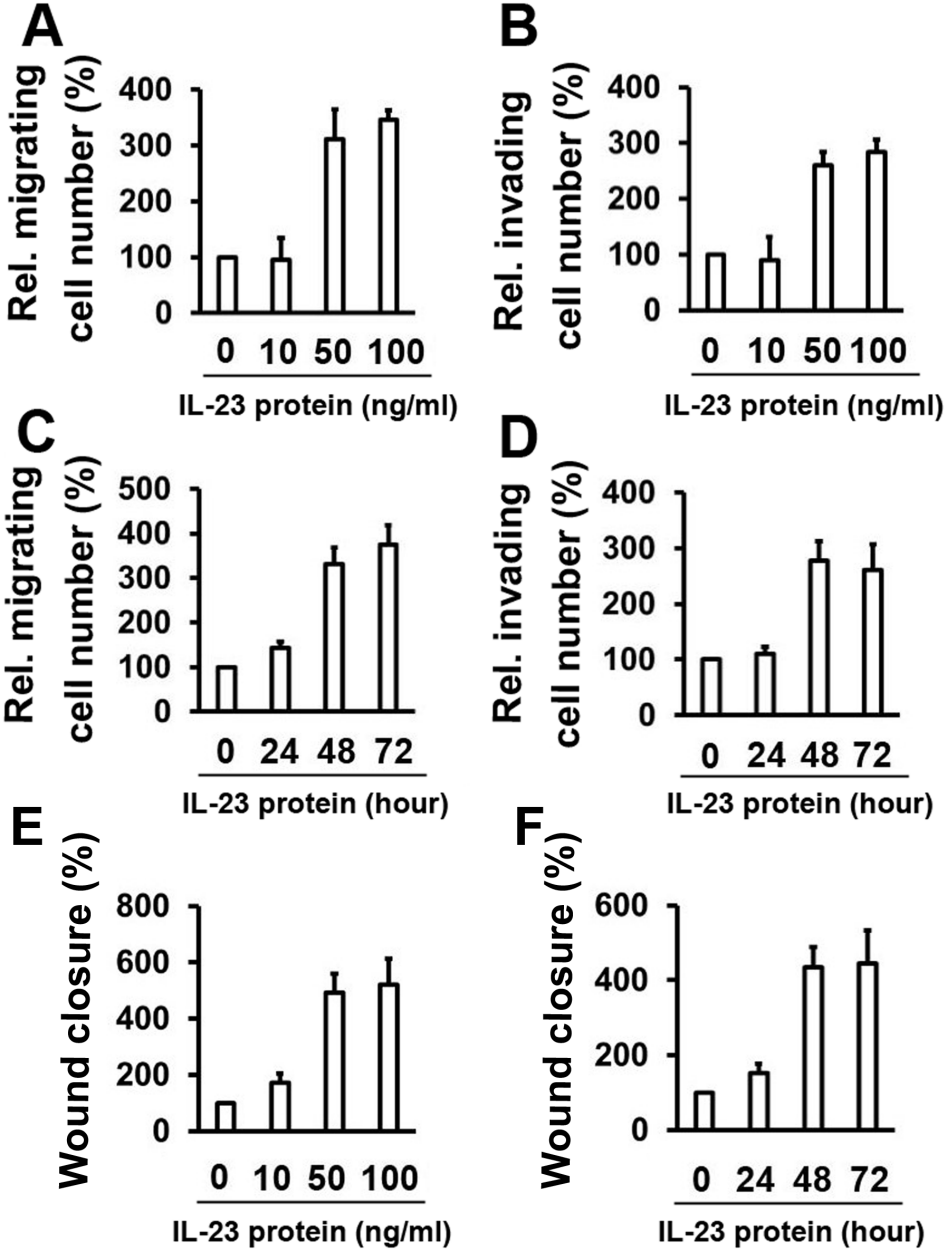
IL–23 promotes K1 cell migration and invasion. (A) K1 cells were treated with rhIL–23 for 48 h at the indicated concentrations, followed by culturing in a transwell system for 24 hours. (B) Cell invasion assay experiments were performed with K1 cells that were treated as in (A). (C) K1 cells were treated with a concentration of 50 ng/ml rhIL–23 for the indicated time points, followed by culturing in a transwell system for 24 h. (D) The experiments were performed as in (C) for the cell invasion assay. (E) K1 cells were treated with rhIL–23 for 48 h at the indicated concentrations, followed by introducing a wound. Cell migration into the wound was monitored at 24 hours. (F) K1 cells were treated with a concentration of 50 ng/ml rhIL–23 for the indicated time points, followed by introducing a wound. Cell migration into the wound was monitored at 24 hours. Wound closure was measured by calculating pixel densities in the wound area and expressed as percentage of wound closure of triplicate areas ± standard deviations In the transwell migration, invasion and wound healing assay, the data are expressed as a percentage of the control. Data represent mean ± SD, n = 3 (**P < 0.01; *P < 0.05).

We wanted to test the effect of IL–23 on the cell proliferation of thyroid cancer cells in order to observe if cell proliferation disturbs the migration and invasion capacity of the cells. The MTT assay showed that the proliferation of K1 cells and WRO cells were hardly affected by any dose of IL–23 (S1 Fig). We next investigated whether IL–23 could affect the migration and the invasion of thyroid cancer cells. Enhanced movement and invasion of K1 cells were detected in the presence of 50 and 100 ng/ml of IL–23 (Fig 1A and 1B). Similarly, increased migration and invasion were also detected as early as 48 hours (h) following treatment with 50 ng/ml IL–23 protein (Fig 1C and 1D). We also demonstrated that IL–23 increased the migration and invasion of WRO cells in a dose- and time-dependent manner (S2 Fig). In addition, wound healing assay also show that IL–23 increased the migration of K1 cells (Fig 1E and 1F). Together, these results confirm the dose- and time-dependent promigratory and proinvasive effect of IL–23.

### IL–23 induces the migration and invasion of thyroid cancer cells through *SOCS4*

**Fig 2:**
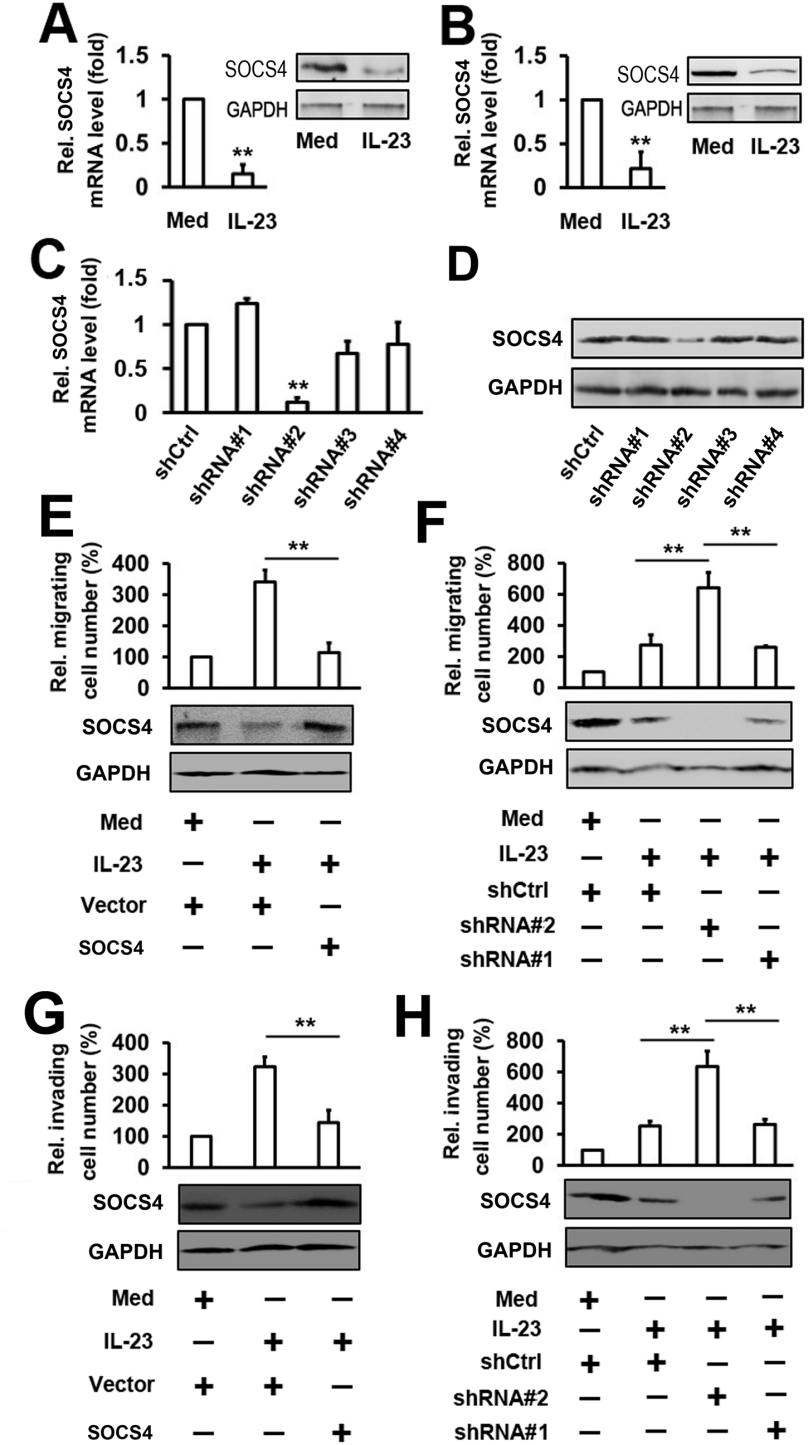
Analysis of the role of So4 in the regulation of thyroid cancer cell migration and invasion mediated by IL–23. (A) K1 cells were treated with 50 ng/ml rhIL–23 for 48 h. *SOCS4* RNA levels were quantified by qRT-PCR (left panel) and the protein levels of SOCS4 were detected by western blot (right panel). (B) Experiments with WRO cells were performed as in A. (C, D) K1 cells were transfected with different *SOCS4* shRNA (shRNA#1, shRNA#2, shRNA#3 and shRNA#4) or shCtrl as mock. After 48 h, *SOCS4* mRNA levels were measured by qRT-PCR (C) and western blot (D). (E) K1 cells were transfected with the indicated plasmid, then treated with rhIL–23 (50 ng/ml) for 48 h and allowed to migrate towards serum for 24 h (upper panel). Protein levels of SOCS4 were detected by western blot (lower panel). (F) The cells were transfected with indicated shRNA-SOCS4 and the experiments were performed as in (E). (G, H) The cell invasion assay experiments were performed under similar conditions as described in (E) and (F). In the real-time RT-PCR experiments, the control was designated as 1. All the experiments were repeated at least 3 times with similar results. Bar graphs represent mean ± SD, n = 3 (**P < 0.01; *P < 0.05).

To identify the role of IL–23 in the expression of SOCS4, K1 cells were stimulated with IL–23 protein for 24 h at the concentration of 50 ng/ml. The suppressors of cytokine signaling (*SOCS4*) mRNA and protein were detected by real-time PCR and western blot, respectively. Results showed that IL–23 suppressed *SOCS4* mRNA and protein expression in K1 cells (Fig 2A). Similar results were also obtained in the WRO cell line (Fig 2B). To determine whether SOCS4 participates in the IL-23-mediated migration and invasion of thyroid cancer cells, we constructed 4 human SOCS4-specific short hairpin RNA (shRNA). Real-time PCR results showed that the #2 shRNA plasmids could markedly inhibit the expression of SOCS4 in K1 cells, whereas the other shRNA plasmids had little effect on the expression of SOCS4 (Fig 2C). SOCS4 protein levels showed a similar trend as determined by Western blot (Fig 2D). In cell migration experiments, the overexpression of *SOCS4* inhibited IL-23-induced migration in K1 cells (Fig 2E). Conversely, the knockdown of *SOCS4* expression enhanced IL-23-induced migration in K1 cells (Fig 2F). The degrees of induction were correlated with the efficiencies of *SOCS4* knockdown by each shRNA plasmid (Fig 2F). Similar results were obtained with transwell invasion experiments (Fig 2G and 2H). Since SOCS4 negatively regulates migration, we next analyzed the role of SOCS4 in the cell proliferation of thyroid cancer cells. Results from MTT assay indicated that the proliferation of K1 cells were hardly affected by the expression of SOCS4. In addition, overexpression or Knockdown of SOCS4 and treatment with IL–23 could not affect the cell proliferation of thyroid cancer cells (S3 Fig). These results suggest that the activation of IL-23-regulated migration and invasion may require SOCS4.

### MicroRNA–25 downregulates *SOCS4* expression by directly targeting its 3′-UTR

To analyze the miRNAs that may target the 3′-UTR of *SOCS4*, we used 3 online databases, TargetScanHuman, miRDB, and miRWalk2.0, to search for potential candidates. Fourteen of the common potential miRNAs were found in the 3 databases. To determine the effect of the predicted miRNAs on the expression of *SOCS4*, the *SOCS4* 3′-UTR was cloned into a firefly luciferase reporter plasmid. The luciferase activity assays indicated that 6 miRNAs inhibited the SOCS4 luciferase activity below 50% as compared with the miR-Ctrl (S4A Fig). We next investigated which miRNAs were activated by IL-23-induced migration and invasion. K1 cells were treated with IL–23 protein at 50 ng/ml for 48 h. Results from real-time PCR demonstrated that among the miRNAs that could attenuate *SOCS4* luciferase activity, the expression of miR–25 was significantly induced (S4B Fig).

**Fig 3:**
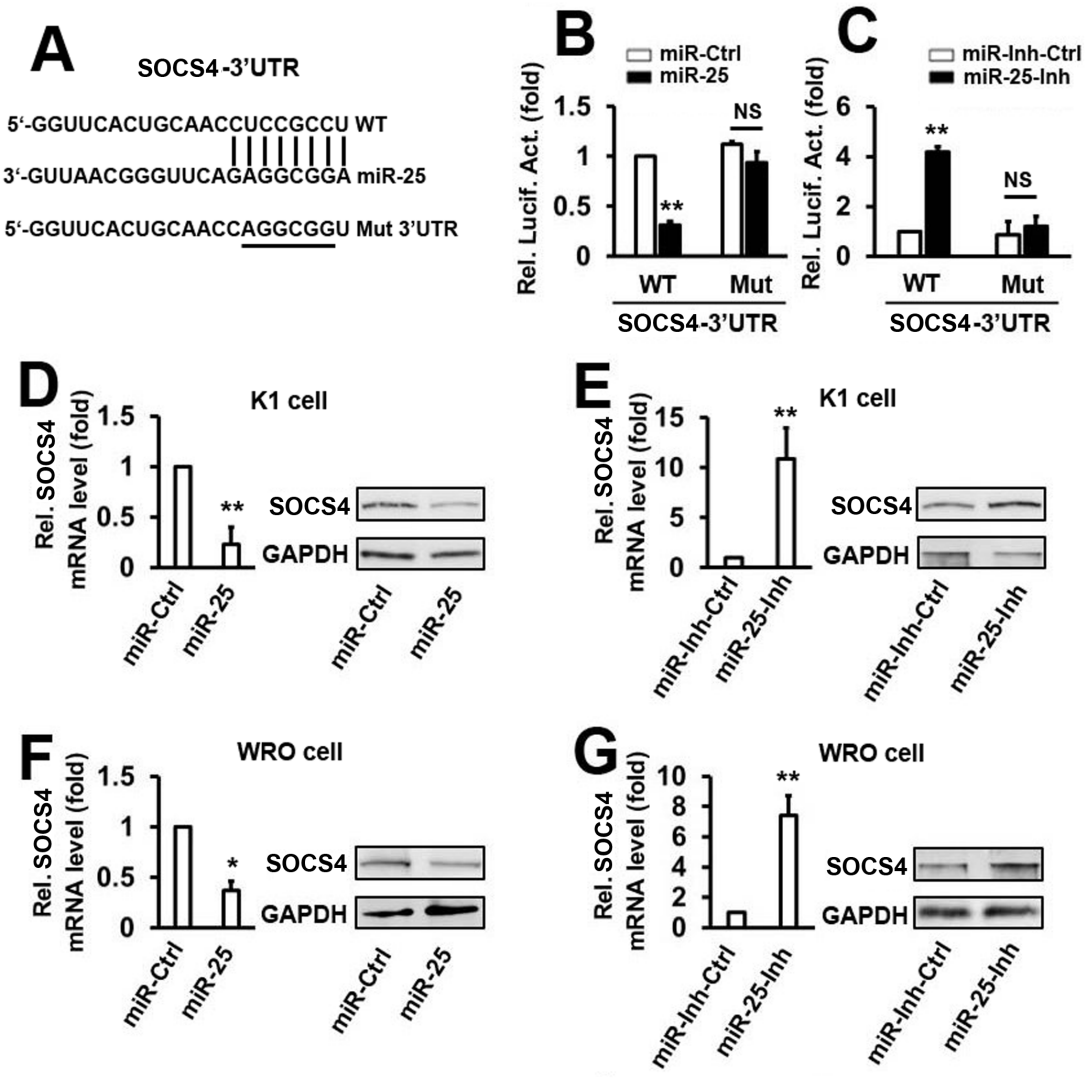
MicroRNA–25 downregulates *SOCS4* expression by directly targeting its 3′UTR. (A) The sequences of miR–25 binding sites within the human *SOCS4* 3′UTRs and the schematic reporter constructs. In this panel, WT represents the reporter constructs containing the entire 3′UTR sequences of *SOCS4*. *SOCS4*-MUT represents the reporter constructs containing mutated nucleotides. (B, C) K1 cells were co-transfected with either WT or MUT *SOCS4* 3′UTR reporter plasmids and either miR–25 mimic (B) or miR–25 inhibitor (C). Luciferase activities were measured after 48 h using a dual-luciferase assay kit and normalized to Renilla luciferase. (D) K1 cells were transfected with miR–25 mimic or controls for 48 h. *SOCS4* mRNA (left panel) and protein (right panel) were detected by qRT-PCR and western blot, respectively. (E) Cells were transfected with miR–25 inhibitor or control and the experiments were performed as in D. (F, G) WRO cells were utilized and experiments were performed as in D and E. All the experiments were repeated at least 3 times with similar results. Bar graphs represent mean ± SD, n = 3 (**P < 0.01; *P < 0.05).

From our data we hypothesized that miR–25 may play a role in the IL–23/SOCS4 signaling pathway. We then investigated whether miR–25 regulates *SOCS4* expression by post-transcriptional targeting of its 3′-UTR. A distinct mutation was generated in the 3′-UTR of *SOCS4* at predicted seed-matching sites to test the interaction between miR–25 and *SOCS4* 3′-UTR (Fig 3A). Transient transfection of K1 cells with wild type (WT) *SOCS4* 3′-UTR, and the miR–25 mimic, leads to a significant decrease in the reporter activity compared with that of the miR-control (Fig 3B). This phenomenon was disrupted when the same cell lines were transfected with *SOCS4* 3′-UTR mutants (Fig 3B). The miR–25 inhibitor (miR-25-Inh) significantly stimulated the luciferase activity of the WT *SOCS4* 3′-UTR, without any effect on Mut *SOCS4* 3′-UTR in K1 cells (Fig 3C). MicroRNA–25 overexpression in K1 cells significantly suppressed both the mRNA and protein levels of SOCS4 (Fig 3D). Conversely, miR–25 inhibitor-mediated knockdown of endogenous miR–25 increased *SOCS4* mRNA and protein expression in K1 cells (Fig 3E). To determine whether the miR-25-mediated downregulation of *SOCS4* expression is a common feature in thyroid cancer cells, similar experiments were performed in WRO cells (Fig 3F and 3G). Together, these results suggest that SOCS4 is a target of miR–25 in K1 cells and WRO cells.

### MicroRNA–25 promotes the motility of thyroid cancer cells by targeting *SOCS4*

**Fig 4:**
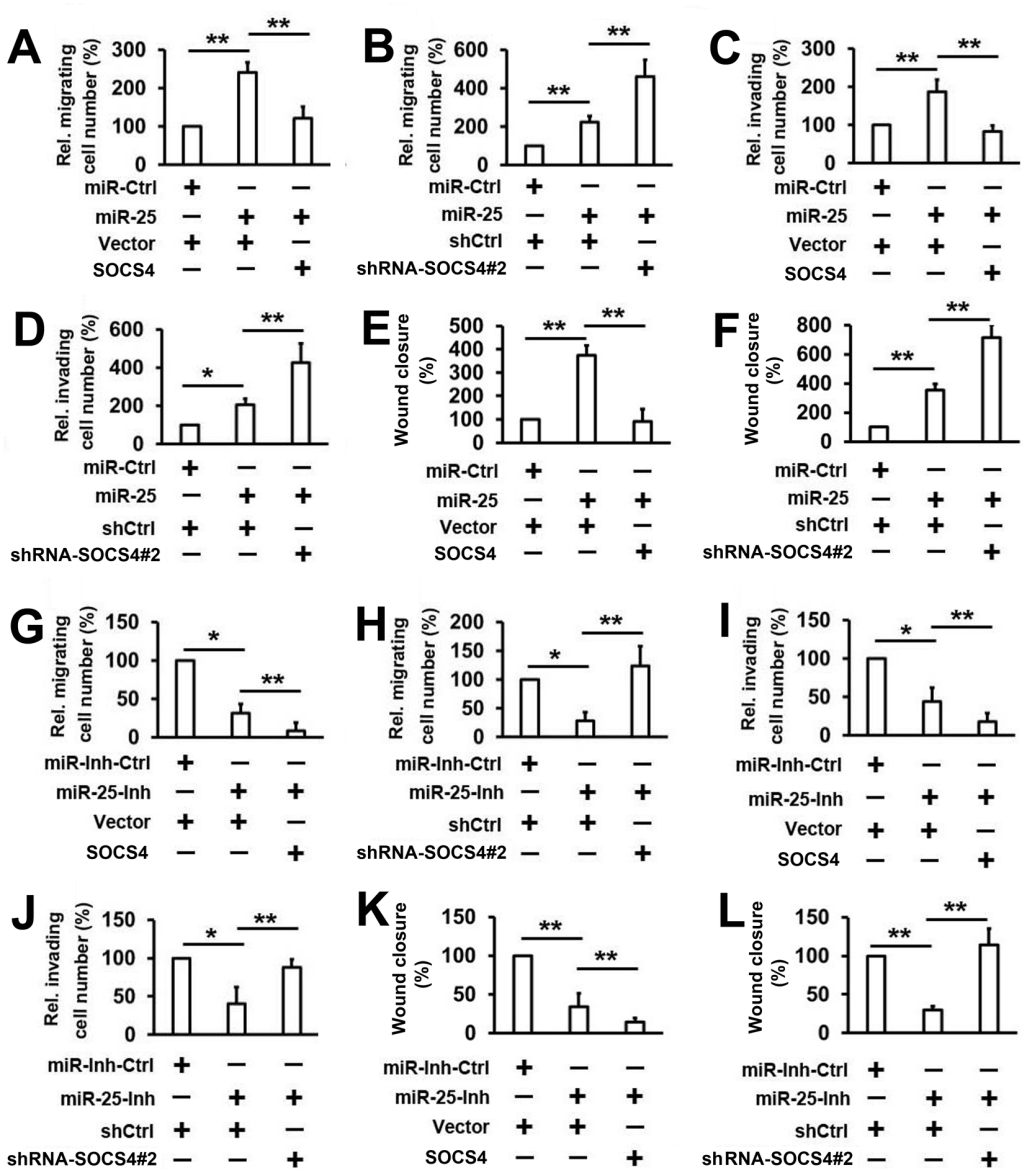
Determination of the effect of miR–25 on the regulation of *SOCS4*-suppressed thyroid cancer cell migration and invasion. (A, B) K1 cells were co-transfected with indicated miR-RNA mimic and plasmid (A) or shRNA (B) for 48 h and allowed to migrate towards serum for 24 h. (C, D) Cell invasion assay experiments were performed with the same conditions as in A and B. (E, F) Wound healing assay experiments were performed with the same conditions as in A and B. (G, H) Cells were transfected with miR–25 inhibitor or control and the experiments were performed as in A and B. (I, J) Cell invasion assay experiments were performed with the same conditions as in G and H. (K, L) Wound healing assay experiments were performed with the same conditions as in G and H. Bar graphs represent mean ± SD, n = 3 (**P < 0.01; *P < 0.05).

Since miR–25 can suppress *SOCS4* expression by sequence-specific binding to its 3′-UTR, we hypothesized that miR–25 may affect the migration and invasion of thyroid cancer via SOCS4. To test the hypothesis, K1 cells were co-transfected with the *SOCS4* expression vector (or empty vector) and the miR–25 mimics (or miR-Ctrl). Transwell migration assays demonstrate that miR–25 promotes the migration of K1 cells and cell migration induced by miR–25 is reversed by *SOCS4* overexpression (Fig 4A). In addition, we observed that the knockdown of *SOCS4* may significantly enhance the effect of miR–25 on cell migration (Fig 4B). Similar results were obtained in the invasion assays and wound healing assay (Fig 4C-4F). The effect of the miR–25/SOCS4 signaling pathway on the migration of thyroid cancer cells was further evaluated using the miR–25 inhibitor. As shown in Fig 4G, miR–25 inhibitor significantly inhibits the migration of K1 cells. In addition, the miR–25 inhibitor and the *SOCS4* expression vector synergistically inhibit the migration of K1 cells (Fig 4G). Conversely, high levels of migration were present in K1 cells when *SOCS4* was knocked down by shRNA-SOCS4#2 (Fig 4H). Similar results were also obtained in invasion assays and wound healing assay (Fig 4I-4L). These results suggest that the inhibition of *SOCS4* expression is responsible for the ability of miR–25 to promote cell invasion and migration.

### IL–23 stimulates miR–25 expression in thyroid cancer cells

**Fig 5:**
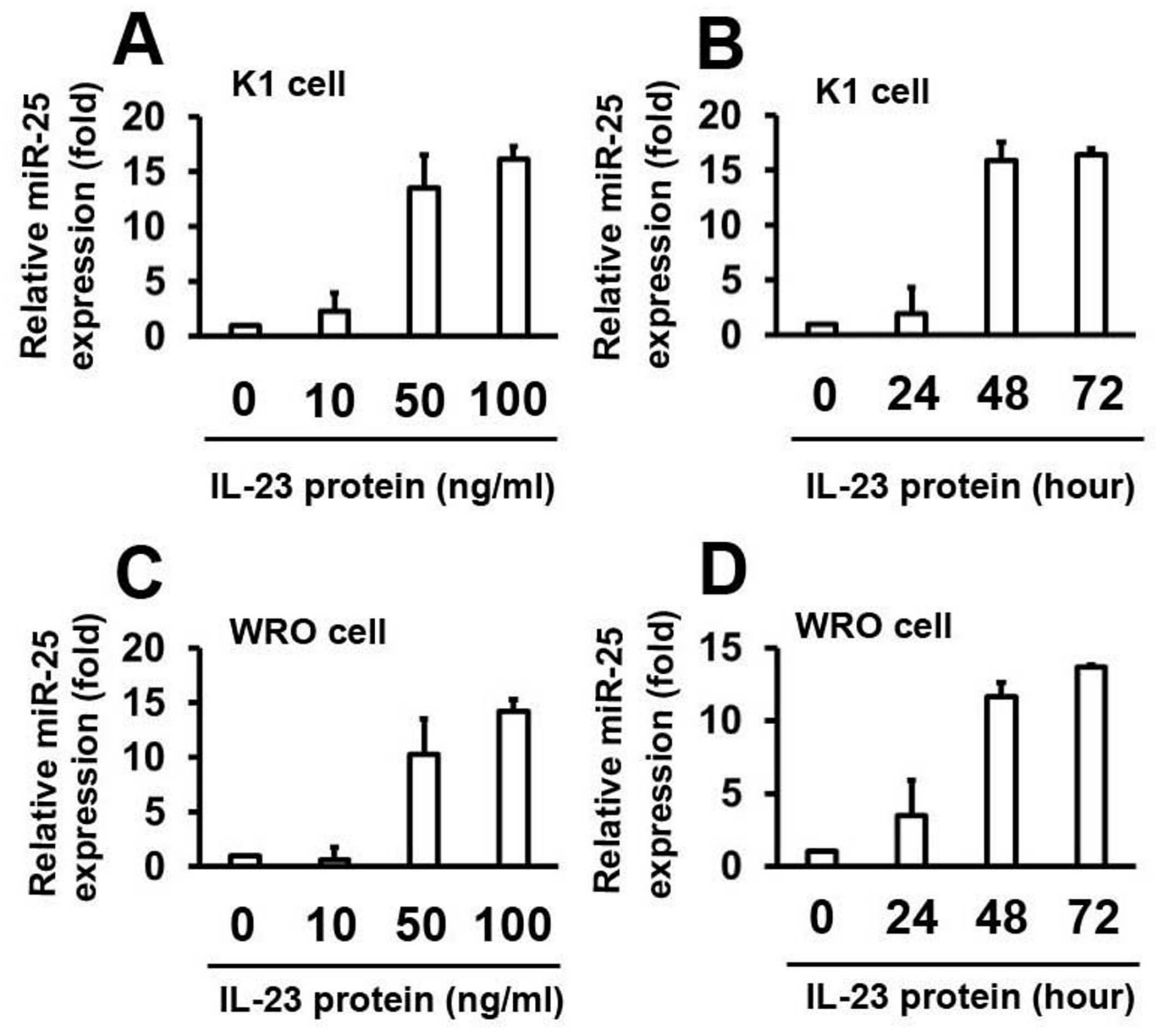
IL–23 stimulates miR–25 expression. MicroRNA–25 levels were quantified by real-time PCR and the experiments were performed as described in Fig 1. The expression levels were normalized to U6 snRNA. Data represent mean ± SD, n = 3 (**P < 0.01; *P < 0.05).

To identify the role of IL–23 in the expression of miR–25, K1 cells were stimulated with human IL–23 protein at different concentrations for 48 h. MicroRNA–25 levels were detected by real-time PCR. Results show that miR–25 levels are upregulated by the IL–23 protein in a dose-dependent manner (Fig 5A). Consistently, K1 cells were treated with IL–23 protein at different time points, at a concentration of 50 ng/ml. Results from real-time PCR analyses demonstrate that miR–25 levels increase as the time increases (Fig 5B). The role of IL–23 on miR–25 expression was confirmed by repeating the experiments using WRO cells (Fig 5C and 5D). These results suggest that IL–23 activates miR–25 expression.

### MicroRNA–25 plays an important role in IL-23-mediated *SOCS4* inhibition and cell migration and invasion

**Fig 6:**
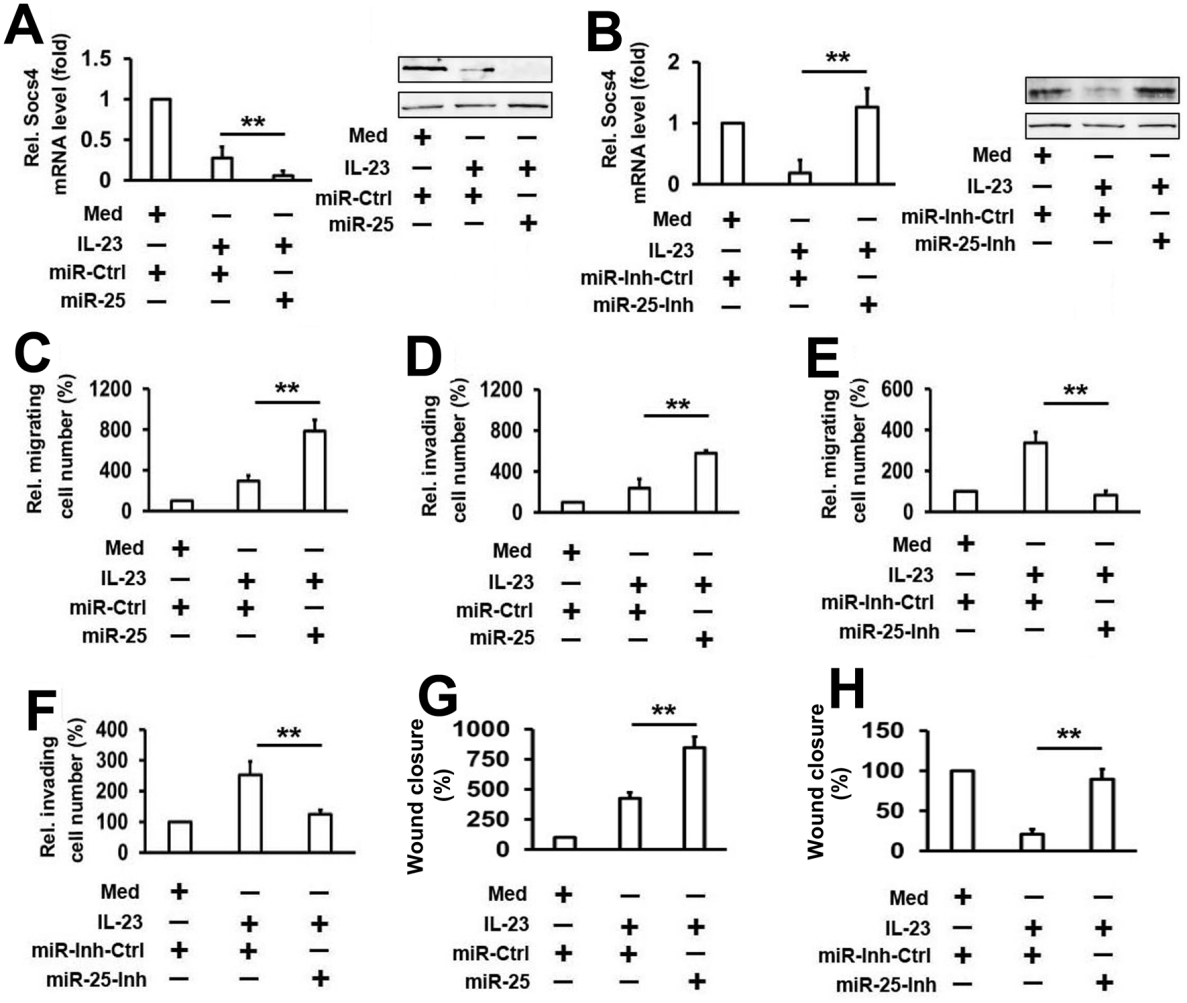
Analysis of the role of miR–25 in the regulation of *SOCS4* expression and IL-23-mediated thyroid cancer cell migration and invasion. (A) K1 cells were transfected with the indicated plasmid, then treated with rhIL–23 (50 ng/ml) for 48 h. SOCS4 mRNA (left panel) and protein (right panel) were detected by qRT-PCR and western blot, respectively. (B) Cells were transfected with miR–25 inhibitor or control, and the experiments were performed as in A. (C, D) K1 cells were transfected with miR–25 mimic (C) or inhibitor (D) and controls, then treated with rhIL–23 (50 ng/ml) for 48 h and allowed to migrate towards serum for 24 h. (E, F) Cell invasion assay experiments were performed with the same conditions as in C and D. (G, H) Wound healing assay experiments were performed with the same conditions as in C and D. All the experiments were repeated at least 3 times with similar results. Bar graphs represent mean ± SD, n = 3 (**P < 0.01; *P < 0.05).

To define the role of miR–25 in the downregulation of IL-23-mediated *SOCS4* expression, K1 cells were transfected with either miR–25 mimics or miR-Ctrl and treated with or without IL–23 protein for 48 h. Results from real-time PCR and western blot analyses show that transfection with miR–25 mimics increases IL-23-mediated inhibition of *SOCS4* mRNA and protein expression (Fig 6A). In contrast, high levels of *SOCS4* mRNA and protein are present in K1 cells, when the expression of miR–25 is inhibited by the miR–25 inhibitor (Fig 6B). We also examined whether miR–25 is involved in IL-23-mediated thyroid cancer cell line motility. Using transwell migration and invasion assays, we showed that miR–25 overexpression stimulates IL-23-mediated activation of migration and invasion (Fig 6C and 6D), and knockdown of miR–25 expression inhibits IL-23-mediated activation of migration and invasion (Fig 6E and 6F). Similar results were also obtained in wound healing assay (Fig 6G and 6H).Taken together, these data suggest that miR–25 is the key component involved in IL-23-mediated thyroid cancer cell migration and invasion through the inhibition of *SOCS4* expression.

### The expression of IL–23, miR–25 and *SOCS4* in thyroid cancer tissues

To validate the role of IL–23, miR–25 and *SOCS4* in thyroid cancer, the expression of IL–23, miR–25 and SOCS4 were analyzed in 35 pairs of clinical PTC, 26 pairs of clinical FTC, and 22 normal thyroid samples. As shown in S5A-S5C Fig, the expression of *SOCS4* was lower and the expression level of IL–23 and miR–25 was higher in PTC and FTC specimens than normal thyroid samples. In addition, high levels of IL–23 were correlated with high levels of miR–25 (S5D Fig). Interestingly, low levels of *SOCS4* expression were correlated with high levels of IL–23 and miR–25 expression in PTC and FTC specimens (S5E and S5F Fig). These observations strongly suggest that alterations of IL–23, miR–25 and SOCS4 expression could be involved in thyroid cancer progression.

## Discussion

In this study, we defined a novel signaling pathway implicated in the control of thyroid cancer cell migration and invasion. First, we demonstrated that IL–23 upregulated miR–25 expression as well as downregulated *SOCS4* expression in thyroid cancer cell migration and invasion. Further, we also showed that miR–25 promotes thyroid cancer cell migration and invasion by targeting SOCS4. Finally, our data suggest that miR–25 is involved in IL-23-associated *SOCS4* expression and cell migration and invasion.

Tumor cell migration and invasion is a very complicated process in which cancer cells spread from the primary tumor, survive in the circulation, and grow in distant locations in the body [28]. Each process is determined by the migration and invasion ability of the tumor cells and the local tumor microenvironment that provide a favorable environment for tumor cells to survive and metastasize [29]. Recent investigations have demonstrated that high expression of levels of IL–23, which can be detected in the microenvironment, could help to facilitate tumor metastasis. For example, IL–23 promotes hepatocellular carcinoma metastasis by NF-κB-upregulated MMP9 expression [9]. IL–23 is highly expressed in metastases-associated astrocytes, and IL–23 induces the progression of melanoma brain metastasis [8]. IL–23 plays a pivotal role in the development of esophageal cancer via an epithelial-mesenchymal transition [7]. IL–23 can enhance the proliferation and invasion of colorectal carcinoma cells [10]. However, the role of IL–23 in thyroid cancer cell migration and invasion is still unknown. To our knowledge, this is the first study to demonstrate the direct effects of IL–23 on the migration and invasion of thyroid cancer cells. Interestingly, a recent study demonstrated that IL–23 regulates the proliferation of lung cancer cells [30]. Nonetheless, unlike lung cancer cells, we did not find evidence to support that IL–23 induces the proliferation of thyroid cancer cells (S1 Fig). We speculate that there may be some intrinsic differences between thyroid cancer and lung cancer. On the other hand, IL–23 promotes the migration and invasion of thyroid cancer cells.

Currently, two reports have implied the potential role of SOCS4 in cancer. One study used a double combination array analysis to prove that SOCS4 is a novel gastric cancer suppressor gene [31]. The other study compared the expression differences of *SOCS1-7* between breast cancer tissue and background breast tissue [32]. High expression of *SOCS4* is significantly associated with an earlier tumor stage and a better clinical outcome in human breast cancer [32]. In this study, we observed that the levels of SOCS4 are decreased in IL-23-induced migration and invasion of thyroid cancer cells (Fig 2). Treatment with IL–23 resulted in reduced expression levels of *SOCS4* in thyroid cancer cells (Fig 2). Through overexpression and knockdown experiments, we demonstrate that *SOCS4* negatively regulates IL-23-induced migration and invasion (Fig 2). Recently, several studies have shown evidence that the SOCS family has a strong tumor suppressing role in several types of solid and hematological tumors [32,33]. When considering the next step, exploring the relationship between the SOCS family and IL–23 could be of great help in further clarifying the role that is played by the SOCS family in the development of thyroid tumors.

The role of miR–25 in cancer is not consistent and is occasionally controversial. In some studies, miR–25 may suppress the proliferation and the migration of colon cancer cells as a tumor suppressor gene *in vitro* and *in vivo* [24,26], whereas in others, miR–25 could remarkably promote cell proliferation and suppress apoptosis in gastric cancer [26]. In this study, we found that miR–25 promotes the migration and invasion of thyroid cancer cells by targeting *SOCS4* (Figs 3 and 4). Additional data reveal that miR–25 is involved in IL-23-regulated cell migration and invasion (Fig 6). These results are consistent with the above studies showing that miR–25 promotes tumor cell migration and invasion. Besides, we also analyzed the role of miR–25 in proliferation, apoptosis, and cell cycle in thyroid cancer cell lines. However, there was no association between miR–25 and cell proliferation, apoptosis, and cell cycle in thyroid cancer cells (data not shown). These results indicate that deregulated miR–25 expression plays different roles in different types of cancers.

In summary, our present study provides a novel evidence indicating that miR–25 and SOCS4 play a functional role in regulating IL-23-mediated migration and invasion in thyroid cancer cells. Although additional studies are required to understand the intricate regulatory mechanisms of thyroid cancer cell migration and invasion, the results obtained may shed new light in understanding cancer resistance to therapy.

## Supporting Information

S1 Fig IL–23 can not affect the proliferation of these thyroid cancer cells. (DOC)

S2 Fig IL–23 promotes the migration and invasion of WRO cell. (DOC)

S3 Fig SOCS4 can not affect the proliferation of these thyroid cancer cells. (DOC)

S4 Fig Screening for miRNAs that are involved in the IL-23-induced signaling pathway and that target the 3’UTR of SOCS4. (DOC)

S5 Fig Expression IL–23, miR–25 and SOCS4 in thyroid cancer tissues. (DOC)

S1 Table Correlation of IL–23, miR–25 and SOCS4 expression with clinicopathologic features in papillary thyroid cancers (PTC). (DOC)

S2 Table Correlation of IL–23, miR–25 and SOCS4 expression with clinicopathologic features in follicular thyroid cancers (FTC). (DOC)

S3 Table Primers Used in Real-time PCR. (DOC)

S4 Table The target sequence of shRNAs. (DOC)
