# Supplementary material for: Interleukin-23 Facilitates Thyroid Cancer Cell Migration and Invasion by Inhibiting SOCS4 Expression via MicroRNA-25
Source: PLoS One. 2015 Oct 5;10(10):e0139456. doi: 10.1371/journal.pone.0139456 (PMC4593557; doi:10.1371/journal.pone.0139456)
Supplement: S1 Fig — (DOC) [file pone.0139456.s001.doc]

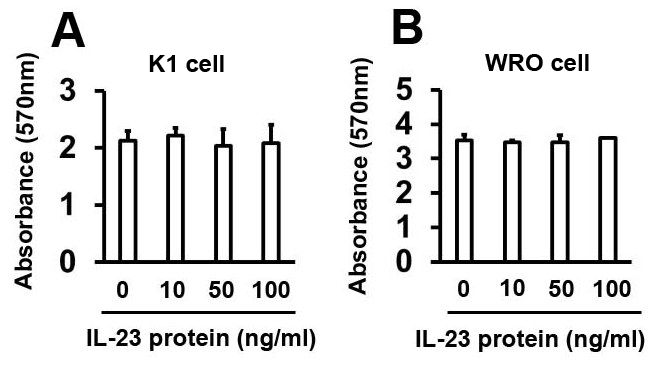


**S1 Fig. IL-23 can not affect the proliferation of these thyroid cancer cells.** K1 cells (A) and WRO cells (B) were treated with rhIL-23 for 48 hour at the indicated concentrations. The proliferation of the cells were quantified by MTT assay.
