# Supplementary material for: Interleukin-23 Facilitates Thyroid Cancer Cell Migration and Invasion by Inhibiting SOCS4 Expression via MicroRNA-25
Source: PLoS One. 2015 Oct 5;10(10):e0139456. doi: 10.1371/journal.pone.0139456 (PMC4593557; doi:10.1371/journal.pone.0139456)
Supplement: S2 Fig — (DOC) [file pone.0139456.s002.doc]

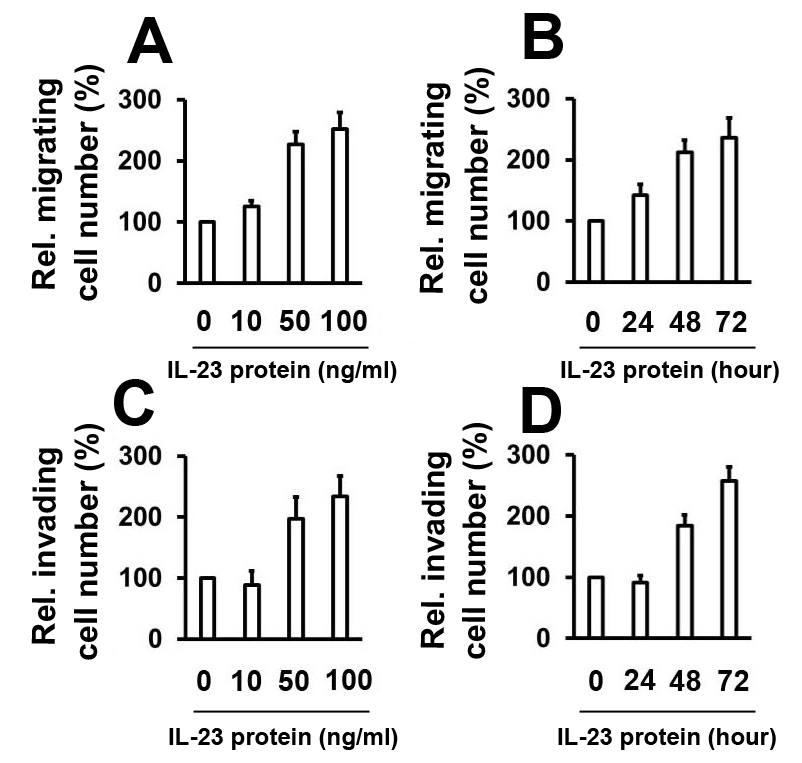


**S2 Fig. IL-23 promotes the migration and invasion of WRO cell.** Experiments were performed as in Fig 1 except WRO cells were performed.
