# Supplementary material for: Interleukin-23 Facilitates Thyroid Cancer Cell Migration and Invasion by Inhibiting SOCS4 Expression via MicroRNA-25
Source: PLoS One. 2015 Oct 5;10(10):e0139456. doi: 10.1371/journal.pone.0139456 (PMC4593557; doi:10.1371/journal.pone.0139456)
Supplement: S3 Fig — (DOC) [file pone.0139456.s003.doc]

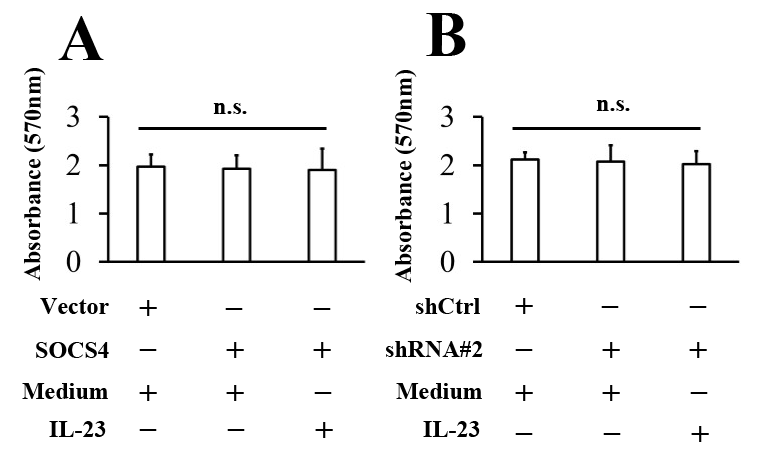


**S3 Fig. SOCS4 can not affect the proliferation of these thyroid cancer cells.** (A) K1 cells were transfected with the indicated plasmid, then treated with rhIL-23 (50 ng/ml) for 48 h. The proliferation of the cells were quantified by MTT assay. (B) The K1 cells were transfected with indicated shRNA-SOCS4 and the experiments were performed as in (A). All the experiments were repeated at least 3 times with similar results.
