# Supplementary material for: Interleukin-23 Facilitates Thyroid Cancer Cell Migration and Invasion by Inhibiting SOCS4 Expression via MicroRNA-25
Source: PLoS One. 2015 Oct 5;10(10):e0139456. doi: 10.1371/journal.pone.0139456 (PMC4593557; doi:10.1371/journal.pone.0139456)
Supplement: S4 Fig — (DOC) [file pone.0139456.s004.doc]

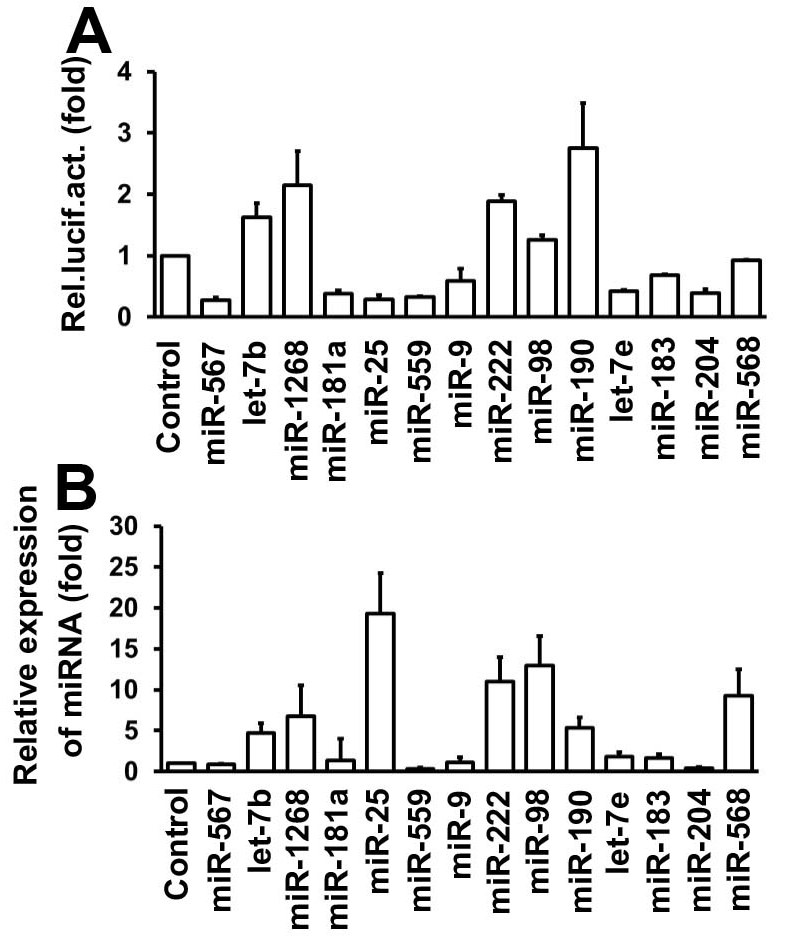


**S4 Fig. Screening for miRNAs that are involved in the IL-23-induced signaling pathway and that target the 3’UTR of SOCS4.** (A) K1 cells were transfected with the indicated miRNA and plasmids for 48 hours prior to luciferase assays. (B) K1 cells were stimulated with 50 ng/ml IL-23 for 48 hours prior to real-time PCR analysis. Experiments were performed three times with similar results.
