# Supplementary material for: Interleukin-23 Facilitates Thyroid Cancer Cell Migration and Invasion by Inhibiting SOCS4 Expression via MicroRNA-25
Source: PLoS One. 2015 Oct 5;10(10):e0139456. doi: 10.1371/journal.pone.0139456 (PMC4593557; doi:10.1371/journal.pone.0139456)
Supplement: S5 Fig — (DOC) [file pone.0139456.s005.doc]

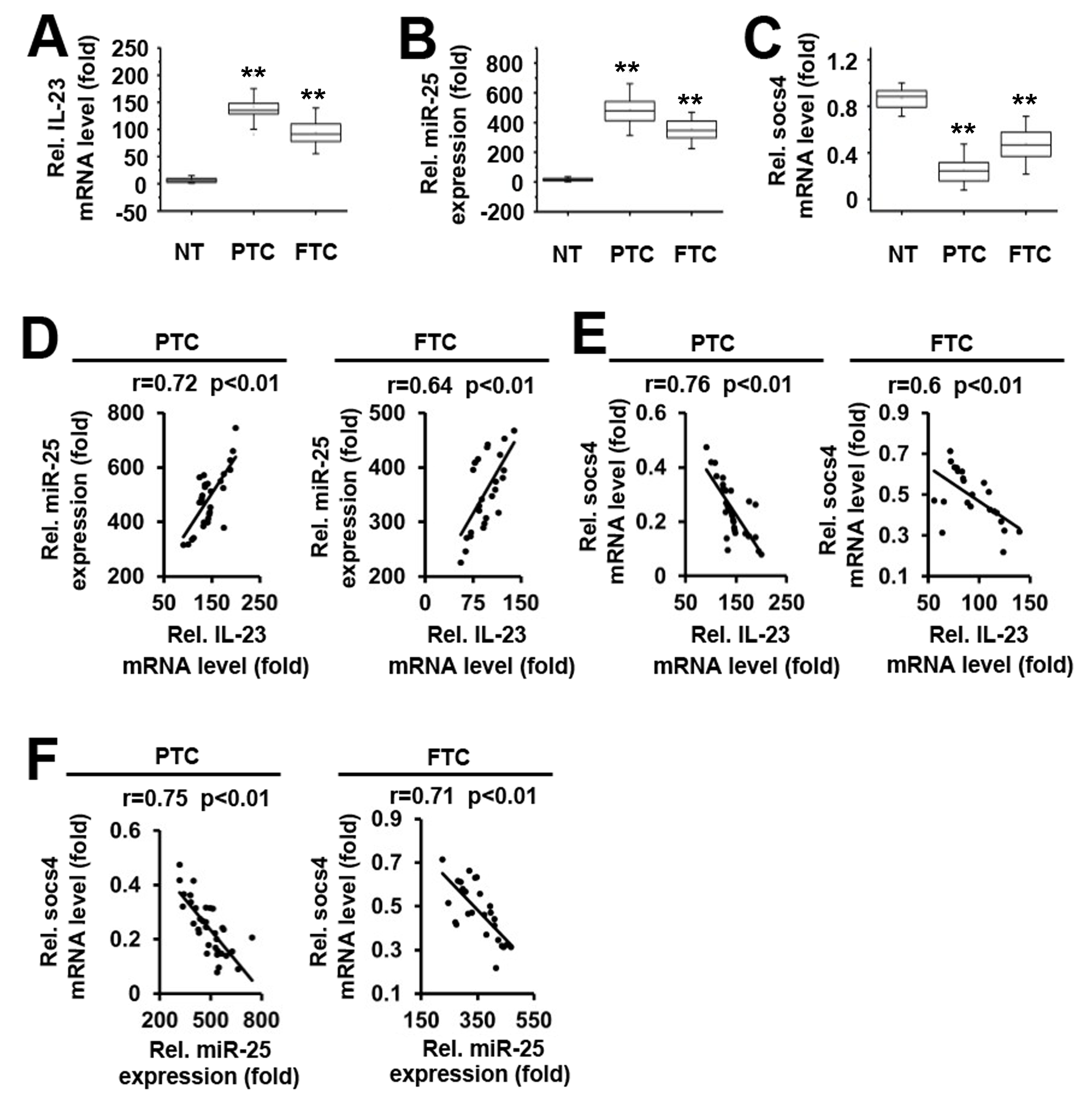


**S5 Fig. Expression IL-23, miR-25 and SOCS4 in thyroid cancer tissues.** (A, B, C) Real-time PCR experiments analyzing the expression of IL-23 (A), miR-25 (B) and SOCS4 (C) in 35 PTCs and 26 FTCs in comparison with mean value of 22 normal thyroid samples (NT). (D) The relative IL-23 mRNA and miR-25 levels in the PTCs (left panel) or FTCs (right panel) were subjected to Pearson’s correlation analysis. (E) The relative IL-23 mRNA and SOCS4 mRNA levels in the PTCs (left panel) or FTCs (right panel) were subjected to Pearson’s correlation analysis. (F) The relative SOCS4 mRNA and miR-25 levels in the PTCs (left panel) or FTCs (right panel) were subjected to Pearson’s correlation analysis. Boxplots illustrate medians with 25% and 75% and error bars for 5% and 95% percentiles. For A-C, the lowest value was designated as 1. IL-23, miR-25 and SOCS4 data are expressed as fold induction (folds) relative to the lowest value (**P < 0.01).
