# Supplementary material for: Interleukin-23 Facilitates Thyroid Cancer Cell Migration and Invasion by Inhibiting SOCS4 Expression via MicroRNA-25
Source: PLoS One. 2015 Oct 5;10(10):e0139456. doi: 10.1371/journal.pone.0139456 (PMC4593557; doi:10.1371/journal.pone.0139456)
Supplement: S1 Table — (DOC) [file pone.0139456.s006.doc]

**Table S1:** Correlation of IL-23, miR-25 and SOCS4 expression with clinicopathologic features in papillary thyroid cancers (PTC).

| **Clinicopathologic parameters** | **Case no.** | **IL-23**  **expression (folds)** | **P value** | **miRNA-25 expression (folds)** | **P value** | **SOCS4**  **expression (folds)** | **P value** |
| --- | --- | --- | --- | --- | --- | --- | --- |
| **Age** |  |  |  |  |  |  |  |
| ≤50 | 20 | 147.6±29.3 | ns | 453.9±67.9 | ns | 0.34±0.12 | ns |
| ＞50 | 15 | 134.8±21.6 |  | 508.7±82.6 |  | 0.21±0.08 |  |
| **Tissue type** |  |  |  |  |  |  |  |
| Normal tissue | 22 | 6.4±4.26 | <0.01 | 16.18±10.67 | <0.01 | 0.87±0.09 | <0.01 |
| Carcinoma | 35 | 142.7±25.9 |  | 485.11±97.53 |  | 0.25±0.1 |  |
| **Sex** |  |  |  |  |  |  |  |
| Male | 19 | 128.9±22.6 | ns | 472.6±71.4 | ns | 0.22±0.05 | ns |
| Female | 16 | 142.3±31.5 |  | 492.6±82.9 |  | 0.28±0.07 |  |
| **Tumor size** |  |  |  |  |  |  |  |
| ≤5cm | 22 | 62.56±9.24 | <0.01 | 280.6±41.6 | <0.01 | 0.41±0.11 | <0.01 |
| ＞5cm | 13 | 224.7±19.4 |  | 682.3±62.3 |  | 0.08±0.01 |  |
| **TNM stage** |  |  |  |  |  |  |  |
| Ⅰand Ⅱ | 20 | 83.6±11.8 | <0.05 | 339.5±25.8 | <0.01 | 0.34±0.12 | <0.05 |
| Ⅲ and Ⅳ | 15 | 199.7±21.9 |  | 640.8±34.8 |  | 0.16±0.05 |  |
| **Lymph nodemetastasis** |  |  |  |  |  |  |  |
| Negative | 23 | 90.36±21.8 | <0.05 | 318.7±35.1 | <0.01 | 0.32±0.14 | <0.05 |
| Positive | 12 | 182.9±17.6 |  | 654.1±28.4 |  | 0.17±0.1 |  |
| **Distant metastasis** |  |  |  |  |  |  |  |
| Negative | 25 | 52.6±9.36 | <0.01 | 219.6±19.5 | <0.01 | 0.43±0.09 | <0.01 |
| Positive | 10 | 237.8±31.5 |  | 750.6±52.8 |  | 0.06±0.01 |  |
