# Supplementary material for: Interleukin-23 Facilitates Thyroid Cancer Cell Migration and Invasion by Inhibiting SOCS4 Expression via MicroRNA-25
Source: PLoS One. 2015 Oct 5;10(10):e0139456. doi: 10.1371/journal.pone.0139456 (PMC4593557; doi:10.1371/journal.pone.0139456)
Supplement: S2 Table — (DOC) [file pone.0139456.s007.doc]

**Table S2:** Correlation of IL-23, miR-25 and SOCS4 expression with clinicopathologic features in follicular thyroid cancers (FTC).

| **Clinicopathologic parameters** | **Case no.** | **IL-23**  **expression (folds)** | **P value** | **miRNA-25 expression (folds)** | **P value** | **SOCS4**  **expression (folds)** | **P value** |
| --- | --- | --- | --- | --- | --- | --- | --- |
| **Age** |  |  |  |  |  |  |  |
| ≤50 | 13 | 90.25±30.2 | ns | 361.3±71.5 | ns | 0.44±0.16 | ns |
| ＞50 | 13 | 97.83±28.3 |  | 349.8±59.5 |  | 0.52±0.24 |  |
| **Tissue type** |  |  |  |  |  |  |  |
| Normal tissue | 22 | 6.4±4.26 | <0.01 | 16.18±10.7 | <0.01 | 0.87±0.09 | <0.01 |
| Carcinoma | 26 | 93.89±21.6 |  | 354.19±68.3 |  | 0.48±0.13 |  |
| **Sex** |  |  |  |  |  |  |  |
| Male | 16 | 87.45±21.6 | ns | 334.8±47.1 | ns | 0.4±0.21 | ns |
| Female | 10 | 100.8±33.6 |  | 371.8±59.2 |  | 0.55±0.25 |  |
| **Tumor size** |  |  |  |  |  |  |  |
| ≤5cm | 14 | 43.9±13.9 | <0.01 | 183.8±20.6 | <0.01 | 0.73±0.15 | <0.01 |
| ＞5cm | 12 | 143.1±22.8 |  | 525.3±67.8 |  | 0.23±0.09 |  |
| **TNM stage** |  |  |  |  |  |  |  |
| Ⅰand Ⅱ | 15 | 67.5±26.9 | <0.05 | 263.4±31.5 | <0.05 | 0.64±0.21 | <0.05 |
| Ⅲ and Ⅳ | 11 | 120.9±35.1 |  | 445.4±58.6 |  | 0.31±0.17 |  |
| **Lymph nodemetastasis** |  |  |  |  |  |  |  |
| Negative | 14 | 60.3±14.9 | <0.01 | 234.5±24.6 | <0.01 | 0.62±0.18 | <0.05 |
| Positive | 12 | 126.7±28.6 |  | 474.2±38.7 |  | 0.33±0.07 |  |
| **Distant metastasis** |  |  |  |  |  |  |  |
| Negative | 17 | 32.6±11.7 | <0.01 | 149.3±17.8 | <0.01 | 0.76±0.24 | <0.01 |
| Positive | 9 | 154.2±37.4 |  | 559.2±63.8 |  | 0.2±0.06 |  |
