# Supplementary material for: Interleukin-23 Facilitates Thyroid Cancer Cell Migration and Invasion by Inhibiting SOCS4 Expression via MicroRNA-25
Source: PLoS One. 2015 Oct 5;10(10):e0139456. doi: 10.1371/journal.pone.0139456 (PMC4593557; doi:10.1371/journal.pone.0139456)
Supplement: S3 Table — (DOC) [file pone.0139456.s008.doc]

Table S4: Primers Used in Real-time PCR

| **Gene name** | **5’primer** | **3’primer** |
| --- | --- | --- |
| **GAPDH** | 5’-GGAAGGTGAAGGTCGGAGTCAACGG-3’ | 5’-CTCGCTCCTGGAAGATGGTGATGGG-3’ |
| **SOCS4** | 5’-CGGGAGGAGTTCATCTGTT-3’ | 5’-GTTTCTTCTGGGCACTTTCT-3’ |
