# Supplementary material for: Interleukin-23 Facilitates Thyroid Cancer Cell Migration and Invasion by Inhibiting SOCS4 Expression via MicroRNA-25
Source: PLoS One. 2015 Oct 5;10(10):e0139456. doi: 10.1371/journal.pone.0139456 (PMC4593557; doi:10.1371/journal.pone.0139456)
Supplement: S4 Table — (DOC) [file pone.0139456.s009.doc]

Table S3: The target sequence of shRNAs

| shRNAs | target sequence (5’ to 3’) |
| --- | --- |
| shRNA-SOCS4#1 | 5’- GAAACAAACCCAAATGGGAT-3’ |
| shRNA-SOCS4#2 | 5’- CATATATGCAGAACAGTTATT-3’ |
| shRNA-SOCS4#3 | 5’- CTGAGACAGTGAATGGTATAG-3’ |
| shRNA-SOCS4#4 | 5’- CCACTTCTATCCACTCCCTTA-3’ |
| shRNA-control | 5’-GTTCTCCGAACGTGTCACGT-3’ |
